# Autophagy-related 7 (ATG7) regulates food intake and liver health during asparaginase exposure

**DOI:** 10.1016/j.jbc.2025.108171

**Published:** 2025-01-10

**Authors:** Brian A. Zalma, Maria Ibrahim, Flavio C. Rodriguez-Polanco, Chintan T. Bhavsar, Esther M. Rodriguez, Eduardo Cararo-Lopes, Saad A. Farooq, Jordan L. Levy, Ronald C. Wek, Eileen White, Tracy G. Anthony

**Affiliations:** 1 Nutritional Sciences Graduate Program, Rutgers University, New Brunswick, New Jersey, United States; 2 Rutgers Cancer Institute of New Jersey, New Brunswick, New Jersey, United States; 3 Department of Nutritional Sciences, Rutgers University, New Brunswick, New Jersey, United States; 4 Department of Molecular Biology and Biochemistry, Rutgers University, Piscataway, New Jersey, United States; 5 Endocrinology and Animal Biosciences Graduate Program, Rutgers University, New Brunswick, New Jersey, United States; 6 Department of Biochemistry and Molecular Biology, Indiana University School of Medicine, Indianapolis, Indiana, United States; 7 New Jersey Institute for Food, Nutrition and Health, Rutgers University, New Brunswick, New Jersey, United States

**Keywords:** eukaryotic initiation factor 2 (eIF2), FGF21, GDF15, translation, gene expression, protein synthesis, amino acid, polysome profiling, body composition

## Abstract

Amino acid starvation by the chemotherapy agent asparaginase is a potent activator of the integrated stress response (ISR) in the liver and can upregulate autophagy in some cell types. We hypothesized that autophagy-related 7 (ATG*7*), a protein that is essential for autophagy and an ISR target gene, was necessary during exposure to asparaginase to maintain liver health. We knocked down *Atg7* systemically (*Atg7*^Δ/Δ^) or in hepatocytes only (ls-*Atg7*KO) in mice before exposure to pegylated asparaginase for 5 days. Intact mice injected with asparaginase lost body weight due to reduced food intake and increased energy expenditure. Systemic *Atg7* ablation reduced liver protein synthesis and increased liver injury in vehicle-injected mice but did not further reduce liver protein synthesis, exacerbate steatosis or liver injury, or alter energy expenditure following 5 days of asparaginase exposure. *Atg7*^Δ/Δ^ mice were unexpectantly protected from asparaginase-induced anorexia and weight loss. This protection corresponded with reduced phosphorylation of hepatic GCN2 and blunted increases in ISR gene targets including growth differentiation factor 15 (GDF15), a negative regulator of food intake. Interestingly, asparaginase elevated serum GDF15 and reduced food intake in ls-*Atg7*KO mice, similar to intact mice. Liver triglycerides and production of the hepatokine fibroblast growth factor 21, another ISR gene target, were suppressed in asparaginase-exposed *Atg7*^Δ/Δ^ and ls-*Atg7*KO mice. This work identifies a bidirectional relationship between autophagy and the ISR in the liver during asparaginase, affecting food intake and liver health.

Asparaginase is an enzyme and key component in the treatment of acute lymphoblastic leukemia (ALL), the most common childhood cancer. Patients undergoing treatment with asparaginase may experience a spectrum of adverse metabolic events that include hepatotoxicity and steatosis (1, 2, 3, 4, 5). The molecular basis for these metabolic complications is not fully understood. Asparaginase depletes circulating asparagine and glutamine, and in the liver activates the highly conserved integrated stress response (ISR) *via* the amino acid starvation sensor named general control nonderepressible 2 (GCN2) (6, 7, 8, 9). In the livers of obese mice, asparaginase also activates the protein kinase R-like endoplasmic reticulum kinase (PERK) (10). GCN2 and PERK phosphorylate the α subunit of eukaryotic initiation factor 2 (eIF2α) to lower global protein synthesis coincident with preferential translation of specific stress response transcription factors such as activating transcription factor 4 (*Atf4*) and CAAT enhancer binding protein homologous protein (CHOP) (11, 12, 13). These and related DNA binding proteins execute a transcriptional program promoting amino acid metabolism and synthesis, antioxidant defenses, autophagy, cell cycle control, and feedback regulation (14). The transcriptional execution of a self-limiting ISR promotes cellular protection, recovery, and adaptation whereas an amplified or sustained ISR triggers cell death. Our laboratory has documented a protective role for the ISR in tissues of mice exposed to asparaginase (9, 15, 16, 17, 18), but how the activation *versus* transcriptional execution of the ISR guides subsequent cellular processes remains unclear.

In both cultured cells and in mouse liver, autophagy is quickly activated upon amino acid starvation (19). Many genes that are involved with autophagosome formation are ISR gene targets regulated by ATF4 and CHOP (20, 21). One of these gene products, autophagy-related 7 (ATG7), is necessary for the lipid conjugation of microtubule-associated protein 1 A/1B-light chain 3 (LC3) to form the autophagosome (20, 22). Systemic ablation of ATG7 renders mice autophagy-deficient and unable to maintain glucose homeostasis during fasting (23). Studies in mice show loss of ATG7 in the liver or whole body slows tumor growth (24). ALL cell lines exposed to asparaginase show that increased autophagic flux is necessary for cell survival (25). Moreover, inhibiting autophagy in ALL cells exposed to asparaginase decreases cell viability (26). These studies collectively suggest that autophagy protects tumors and body cells against the stress of amino acid insufficiency induced by asparaginase. However, the hypothesis that autophagy functions to maintain liver health during asparaginase exposure has never been tested directly.

This study explored the role of autophagy in regulating whole-body metabolism and liver proteostasis following exposure to asparaginase. To accomplish this, we administered pegylated asparaginase (PEG-asparaginase) to mice with a systemic or liver-specific deletion of *Atg7*. We hypothesized that an inability to initiate autophagy would amplify amino acid stress induced by PEG-asparaginase and challenge the cytoprotective ISR. To our surprise, we found that global loss of *Atg7* during exposure to PEG-asparaginase impaired transcriptional execution of the ISR in the liver and protected mice from weight loss and liver steatosis. Mice with a liver-specific deletion of *Atg7* were also protected from PEG-asparaginase-induced weight loss and displayed a blunted transcriptional execution of the ISR in the liver alongside reduced steatosis. Taken together, these data uncover a bidirectional relationship between the ISR and autophagy.

## Results

### Loss of *Atg7* protects mice from PEG-asparaginase-induced weight loss

Previously, our lab utilized the native form of asparaginase (Elspar) to examine the physiological and mechanistic responses to asparaginase. The short half-life of this formulation required us to administer the drug every 24 h (9) and the frequency of administration precluded the use of indirect calorimetry to measure metabolism over time. In this study, we used PEG-asparaginase which has a much longer half-life in both humans and mice, allowing for a single-dose study design to assess longer-term outcomes (27, 28). Kumar and colleagues exposed mice to a single dose of PEG-asparaginase at 1.5 IU per g body weight and monitored liver health over 7 days (29). That study reported maximum PEG-asparaginase-associated hepatotoxicity between 3 to 5 days after injection. Based on these observations, we compared body weight changes and ISR activation in the liver of mice at 5 days after a single IP injection of PEG-asparaginase at a dose that matched our previous work using Elspar (3.0 IU/g body weight) (18) *versus* the lower dose of 1.5 IU/g body weight. Both doses of PEG-asparaginase similarly induced a significant (∼15%) amount of body weight over the course of the 5-day study period (Fig. S1*A*). Additionally, phosphorylation of eIF2α in the liver was elevated by both doses compared to phosphate-buffered saline (PBS) controls (Fig. S1*B*). These results prompted us to inject mice with 1.5 IU/g body weight PEG-asparaginase for the remaining experiments.

**Figure 1:**
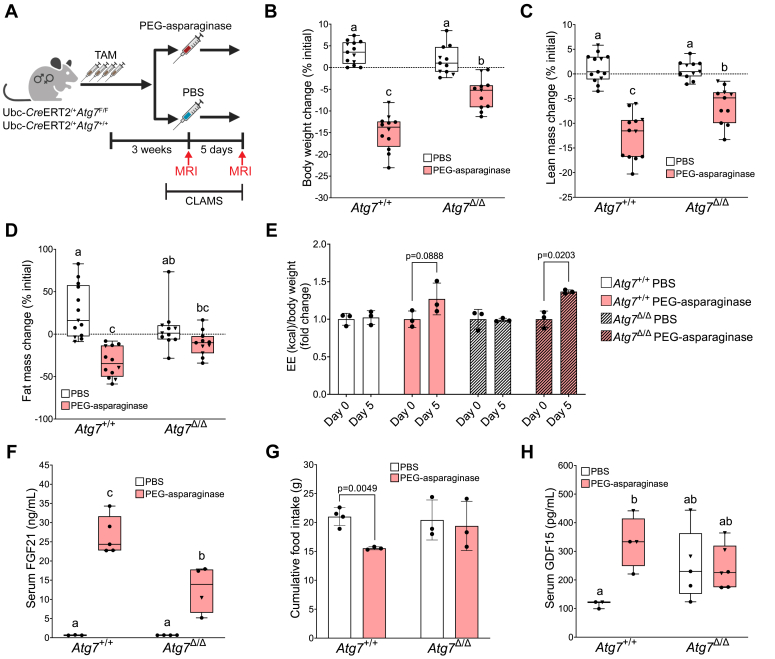
**Knockdown of *Atg7* blunts PEG-asparaginase induced weight loss.***A*, animal experimental design. Male and female *Atg7*^+/+^ and *Atg7*^F/F^ mice at 8 weeks of age were IP injected with tamoxifen once daily for 4 days. Three weeks later, *Atg7*^+/+^ and *Atg7*^Δ/Δ^ mice were IP injected once with PEG-asparaginase (1.5 IU/g) or equivolume PBS as vehicle and sacrificed 5 days later. n = 48 total (3–4 males, 6–7 females, per group). *B*, the difference between body weight at the time of PEG-asparaginase injection (start) and end of the experiment (% initial). n = 47 total (3–4 males, 6–7 females, per group). *C*, the difference between lean body mass at the start and end of the experiment (% initial) as determined by MRI. n = 47 total (3–4 males, 6–7 females, per group). *D*, the difference between body fat mass at the start and end of the experiment (% initial) as determined by MRI. n = 47 total (3–4 males, 6–7 females, per group). *E*, energy expenditure of *Atg7*^+/+^ and *Atg7*^Δ/Δ^ mice over a 24 h period, normalized to body weight, at the start (Day 0) and end (Day 5) of the study, expressed as fold change of each group’s mean Day 0 value. n = 3 (female) per group. Differences in group means were analyzed by repeated measures ANOVA with a Šídák’s multiple comparison’s *post hoc* test. *F*, serum FGF21 concentrations at the end of the study. n = 16 total (15 females, 1 male). *G*, cumulative food intake over the 5-day study period. n = 13 total (13 females). A Welch’s *t* test was performed to compare group means. *H*, serum GDF15 concentrations at the end of the study. n = 18 total (8 males, 10 females). Box plots show median values, *top* and *bottom* hinges refer to the first and third quartiles (25th and 75th percentiles), and the ends of the whiskers mark the smallest and largest values. Bar charts show mean values ± SD. • = females. ▼ = males. Unless otherwise stated, data in each graph were analyzed by a two-way ANOVA (genotype x drug). If a statistical interaction occurred, groups not sharing a common letter indicated a statistically significant difference between groups after *post hoc* pairwise comparisons were conducted with a Tukey correction for multiple comparisons. Significance threshold = *p* ≤ 0.05.

To elucidate the role of autophagy in the homeostatic response to PEG-asparaginase, we administered PEG-asparaginase or PBS as vehicle control by IP injection to *Atg7*^+/+^ and *Atg7*^Δ/Δ^ mice 3 weeks post-tamoxifen exposure (Fig. 1*A*). Previous reports describing *Atg7*^Δ/Δ^ mice at this time point show they express little to no ATG7 protein in the liver and other tissues and are susceptible to fasting-induced hypoglycemia, but do not yet show signs of wasting or severe neurodegeneration (23, 24). Body composition was analyzed by MRI (EchoMRI) at the time of injection and prior to euthanasia. There were no significant differences in average body weight and lean mass in *Atg7*^+/+^ and *Atg7*^Δ/Δ^ mice prior to administration of PEG-asparaginase or PBS (Fig. S2, *A* and *B*). *Atg7*^Δ/Δ^ mice had slightly more fat mass relative to body weight compared to *Atg7*^+/+^ mice prior to injections (Fig. S2*C*). Effective knockdown of the ATG7 protein in both the liver and kidney was confirmed by immunoblot (Fig. S2*D*). Likewise, LC3-I and p62 were substantially elevated in the livers of *Atg7*^Δ/Δ^ mice (Fig. S2, *G* and *H*). All mice were euthanized 5 days after administration of PEG-asparaginase or PBS vehicle. (Fig. 1*A*). PEG-asparaginase induced an approximate 15% decrease in body weight in *Atg7*^+/+^ mice as compared to PBS controls (Fig. 1*B*). However, *Atg7*^Δ/Δ^ mice exposed to PEG-asparaginase lost significantly less body weight, only ∼5% as compared to PBS controls (Fig. 1*B*). Body composition analysis *via* MRI showed that the mitigated weight loss in *Atg7*^Δ/Δ^ mice exposed to PEG-asparaginase reflected retention of both lean and fat mass (Fig. 1, *C* and *D*). PEG-asparaginase reduced adipose tissue weight in both epididymal and inguinal fat pads (Fig. S3, *A* and *B*). To pinpoint the reason for the differences in body weight and composition in response to PEG-asparaginase, we placed a subset of *Atg7*^+/+^ and *Atg7*^Δ/Δ^ mice into the Oxymax CLAMS system to measure energy expenditure and food intake. Although *Atg7*^Δ/Δ^ mice exposed to PEG-asparaginase displayed the highest absolute energy expenditure (Fig. S4, *A* and *B*), PEG-asparaginase increased energy expenditure relative to body weight similarly in both genotypes (Fig. 1*E*). In alignment with this increase, serum concentrations of FGF21, a hormone that is elevated by asparaginase (18) and corresponds with increased energy expenditure in other models of amino acid insufficiency (30, 31, 32) were significantly increased in all PEG-asparaginase-treated mice, although *Atg7*^Δ/Δ^ mice showed lower serum FGF21 concentrations as compared to *Atg7*^+/+^ mice (Fig. 1*F*). PEG-asparaginase did not significantly alter the respiratory exchange ratio in mice of either genotype (Fig. S4, *C* and *D*). Total activity, measured as total XYZ beam breaks, was lower in *Atg7*^Δ/Δ^ mice, consistent with the expected phenotype (23) (Fig. S4*E*). Also, while in the CLAMS, the cages housing *Atg7*^Δ/Δ^ mice recorded significantly less food intake as compared to *Atg7*^+/+^ mice, independent of treatment (Fig. S4*F*). At the end of the CLAMS experiment, we noted unaccounted differences in food spillage. This motivated us to measure food intake manually, housing mice in wire-bottom cages while blinded to genotype identity to properly control for this variable. When accounting for spillage, food intake measurements showed that PEG-asparaginase lowered food intake in *Atg7*^+/+^ mice but not *Atg7*^Δ/Δ^ mice (Fig. 1*G*). We then measured serum concentrations of growth differentiation factor 15 (GDF15), a hormone that is increased during amino acid insufficiencies and suppresses food intake in rodents (33, 34). Serum concentrations of GDF15 increased in PEG-asparaginase-exposed *Atg7*^+/+^ mice (Fig. 1*H*). While circulating GDF15 was elevated basally in *Atg7*^Δ/Δ^ mice, PEG-asparaginase did not further increase serum GDF15 above PBS-exposed *Atg7*^Δ/Δ^ mice (Fig. 1*H*). These results suggest that early loss of *Atg7* rescues the suppression in food intake during PEG-asparaginase exposure, preventing weight loss.

### Autophagy is required to maintain basal translation in the liver

**Figure 2:**
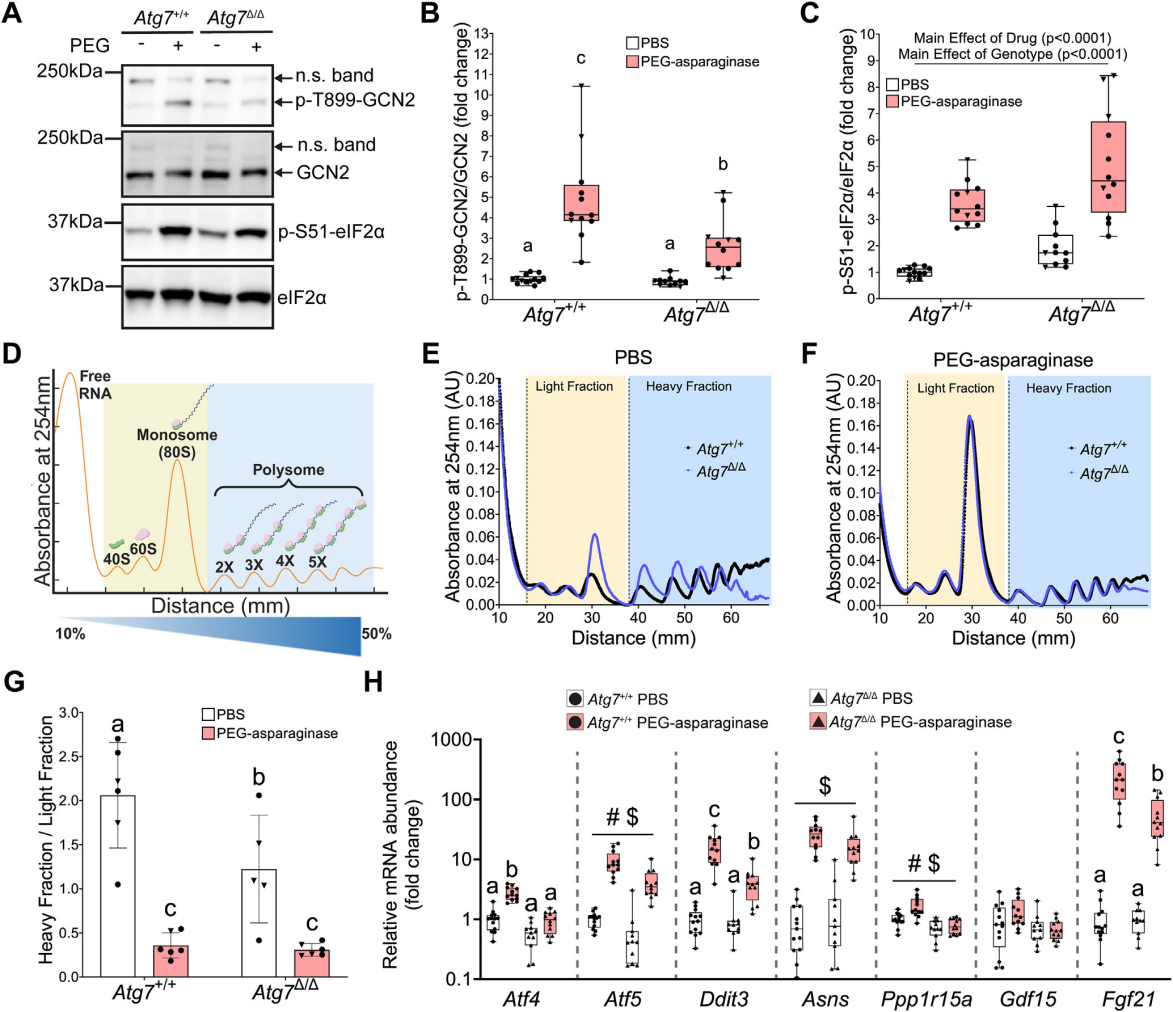
**Whole-body knockdown of *Atg7* modulates the ISR in the liver.***A*, representative immunoblots of p-T899-GCN2/total GCN2 and p-S51-eIF2α/total eIF2α. *B*, quantification of p-T899-GCN2/total GCN2 expressed as fold change of PBS-treated *Atg7*^+/+^ mice. n = 48 total (3–4 males, 6–7 females, per group). *C*, quantification of p-S51-eIF2α/total eIF2α expressed as fold change of PBS-treated *Atg7*^+/+^ mice. n = 48 total (3–4 males, 6–7 females, per group). *D*, graphical representation of polysome profile results. *E*, representative polysome profiles in the livers of *Atg7*^+/+^ and *Atg7*^Δ/Δ^ mice administered PBS. *F*, representative polysome profiles in the livers of *Atg7*^+/+^ and *Atg7*^Δ/Δ^ mice administered PEG-asparaginase. *G*, the absorbance spectrum area under the curve was measured and the ratio of heavy fraction area (*i.e.*, polysomes) over light fraction area (*i.e.*, monosomes and free ribosomal subunits) was calculated and compared across experimental groups. n = 23 total (9 males, 14 females). *H*, mRNA abundance of ISR target genes analyzed by qPCR. n = 48 total (3–4 males, 6–7 females, per group). Box plots show median values, *top* and *bottom* hinges refer to the first and third quartiles (25th and 75th percentiles), and the ends of the whiskers mark the smallest and largest values. Panels (*B*), (*C*), (*G*): • = females. ▼ = males. Bar charts in panels *G* and *H* show mean values ± SD. Data in each graph were analyzed by a two-way ANOVA (genotype x drug). # represents a significant main effect of genotype. $ represents a significant main effect of the drug. If a statistical interaction occurred, groups not sharing a common letter indicated a statistically significant difference between groups after *post hoc* pairwise comparisons were conducted with a Tukey correction for multiple comparisons. Significance threshold = *p* ≤ 0.05.

FGF21 and GDF15 are both gene targets inclusive of the transcriptional response executed upon ISR activation. To understand the effect of *Atg7* loss on the ISR in the liver, we first measured the activation of GCN2 and PERK, regulators of eIF2 phosphorylation. Similar to the observed serum FGF21 pattern, phosphorylation of GCN2 was significantly increased in all PEG-asparaginase-treated mice, although *Atg7*^Δ/Δ^ mice were blunted as compared to *Atg7*^+/+^ mice (Fig. 2, *A* and *B*). On the other hand, PERK phosphorylation was not detected in the livers of mice in any groups (Fig. S5).

We next examined the phosphorylation of eIF2α and observed a main effect of the drug, supporting GCN2 activation. Further, eIF2α phosphorylation was elevated in *Atg7*^Δ/Δ^ mice both basally and following exposure to PEG-asparaginase (Fig. 2, *A* and *C*).

We next examined general translation by conducting the polysome profiling technique on liver samples (Fig. 2*D*). Following ultracentrifugation of liver samples on a 10 to 50% sucrose density gradient, spectrophotometric absorbance at 254 nm was continuously monitored across the sucrose gradient as a proxy for ribosomes. The area of absorbances representing translationally active ribosomes (multiple 80S ribosomes bound to mRNA known as polysomes, labeled heavy fraction) was then calculated as a ratio over the area representing translationally inactive ribosomes (40S and 60S ribosomal subunits free or bound to RNA, plus the 80S monosome, labeled light fraction). In the vehicle PBS condition, global loss of *Atg7* reduced the ratio of polysomes (heavy fraction area) to monosomes plus free ribosomes (light fraction area), indicating lowered protein synthesis (Fig. 2, *E* and *G*). PEG-asparaginase further reduced protein synthesis independent of genotype (Fig. 2, *F* and *G*). These data indicate that loss of *Atg7* decreases general protein synthesis in the liver basally but does not further repress liver translation during PEG-asparaginase exposure.

### PEG-asparaginase-induced transcripts of stress response genes are blunted in autophagy-deficient mice

A key feature of the ISR is preferential translation of *Atf4* which functions to execute an altered transcriptional program that first supports homeostatic recovery, then later programmed cell death if the stress is unrecoverable (13). qPCR analysis of liver samples showed that global loss of *Atg7* blunted the PEG-asparaginase-induction of ISR target genes, *Atf4*, *Atf5*, DNA damage-inducible 3 (*Ddit3*; also known as CHOP), protein phosphatase 1 regulatory subunit 15A (*Ppp1r15a*), *Gdf15*, and *Fgf21* (Fig. 2*H*). Transcript levels of asparagine synthetase (*Asns*) were unaffected by genotype (Fig. 2*H*). Furthermore, the pattern of hepatic *Gdf15* and *Fgf21* mRNA abundances agreed with measured serum concentrations. These results indicate that *Atg7* is necessary for full ISR transcriptional execution during PEG-asparaginase exposure.

### Whole-body loss of *Atg7* induces hepatoxicity

**Figure 3:**
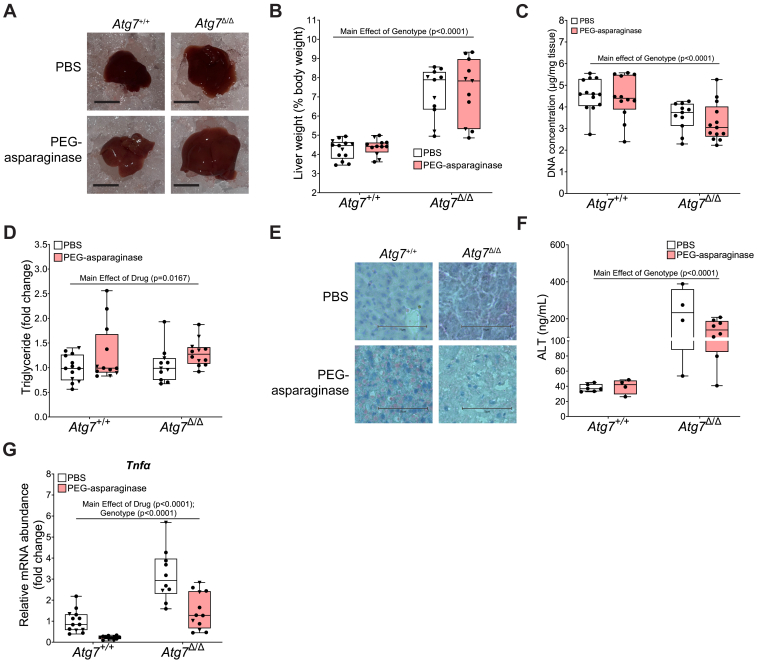
**Systemic reduction of *Atg7* does not worsen liver health during PEG-asparaginase exposure.***A*, whole livers from *Atg7*^+/+^ and *Atg7*^Δ/Δ^ mice exposed to PEG-asparaginase or PBS vehicle. Scale bar is 1 cm. *B*, liver weight expressed as percent body weight. n = 47 total (3–4 males, 6–7 females, per group). *C*, hepatic DNA concentration. n = 48 total (3–4 males, 6–7 females, per group. *D*, liver triglycerides normalized to DNA content, expressed as fold change of *Atg7*^+/+^, PBS-treated mice. n = 48 total (3–4 males, 6–7 females, per group). *E*, Oil Red O and hematoxylin staining on representative liver sections of *Atg7*^+/+^ and *Atg7*^Δ/Δ^ mice exposed to PEG-asparaginase or PBS control. The scale bar is 75 μM. *F*, serum concentrations of ALT at end of the experiment. n = 23 total (23 females). *G*, hepatic mRNA abundance of *Tnfα* analyzed by qPCR. n = 48 total (3–4 males, 6–7 females, per group). Box plots show median values, *top* and *bottom* hinges refer to the first and third quartiles (25th and 75th percentiles), and the ends of the whiskers mark the smallest and largest values. • = females. ▼ = males. Data in each graph were analyzed by a two-way ANOVA (genotype x drug). Significance threshold = *p* ≤ 0.05.

PEG-asparaginase causes hepatic steatosis and hepatotoxicity in humans and mice (1, 35, 36, 37). *Atg7*-deficient mice have exacerbated hepatic steatosis during fasting (38). In line with the reported phenotype of *Atg7* deficiency, *Atg7*^Δ/Δ^ mice exhibited enlarged livers (Fig. 3, *A* and *B*). Additionally, average liver liver DNA concentrations were significantly reduced in *Atg7*^Δ/Δ^ mice (Fig. 3*C*). In contrast, PEG-asparaginase did not reduce liver DNA concentrations in either *Atg7*^+/+^ or *Atg7*^Δ/Δ^ mice. To understand the effect of *Atg7* on liver dysfunction, we measured hepatic triglyceride content and observed mild elevations in steatosis with PEG-asparaginase exposure, although this result did not reach statistical significance (Fig. 3*D*). However, Oil Red O staining of liver sections showed lipid accumulation in the livers of PEG-asparaginase-exposed *Atg7*^+/+^ but not *Atg7*^Δ/Δ^ mice (Fig. 3*E*). We next measured serum concentrations of alanine aminotransferase (ALT), a marker for hepatotoxicity. We found *Atg7*^Δ/Δ^ mice had significantly higher serum ALT concentrations compared to *Atg7*^+/+^ mice (Fig. 3*F*). PEG-asparaginase exposure did not increase circulating ALT in *Atg7*^+/+^ mice, nor was circulating ALT further increased in *Atg7*^Δ/Δ^ mice (Fig. 3*F*). Previous work from our lab showed that asparaginase induces hepatic inflammation in mice deficient in GCN2 (9). We measured mRNA transcript abundance of the inflammatory cytokine, tumor necrosis factor α (*Tnfα*) to test if autophagy protects mice against PEG-asparaginase-induced inflammation. We observed that *Atg7*^Δ/Δ^ mice had significantly greater hepatic *Tnfα* transcript abundance relative to *Atg7*^+/+^ mice and that PEG-asparaginase decreased levels independent of genotype (Fig. 3*G*). We interpret these data to suggest that opposite to our original hypothesis, early systemic loss of *Atg7* does not exacerbate liver health during PEG-asparaginase exposure.

### Hepatic *Atg7* is required for body weight and lean mass loss in response to PEG-asparaginase

**Figure 4:**
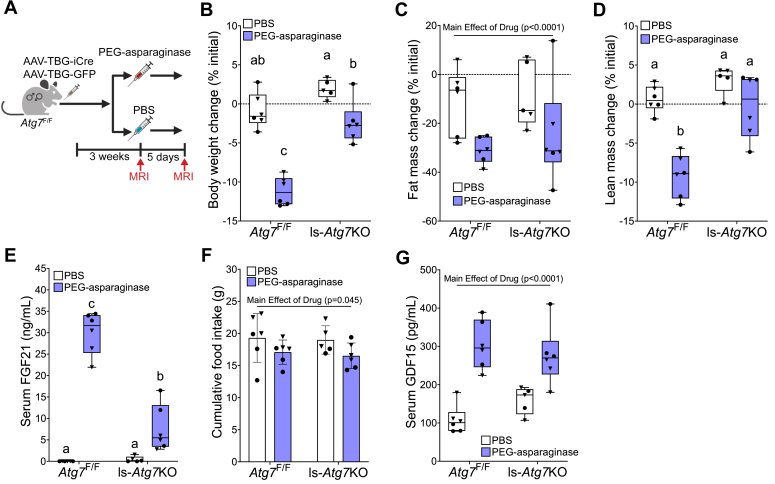
**Liver-specific knockdown of *Atg7* protects mice from PEG-asparaginase-induced weight loss.***A*, animal experimental design. Male and female *Atg7*^F/F^ mice at 8 weeks of age were tail vein injected with AAV-TBG-iCre (ls-*Atg7*KO mice) or AAV-TBG-GFP as control (*Atg7*^F/F^). Three weeks after injection, mice were IP injected once with PEG-asparaginase (1.5 IU/g) or equivolume PBS as a vehicle. n = 23 (2–3 males, 2–3 females, per group). *B*, difference between body weight at the time of PEG-asparaginase injection (start) and end of the experiment (% initial). n = 23 (12 males, 11 females). *C*, the difference between body fat mass at the start and end of the experiment (% initial) as determined by MRI. n = 23 (12 males, 11 females). *D*, the difference between lean body mass at the start and end of the experiment (% initial) as determined by MRI. n = 23 (12 males, 11 females). *E*, serum concentrations of FGF21 at the end of the experiment. n = 23 (12 males, 11 females). *F*, cumulative food intake (g) between the start and end of the experiment. n = 23 (12 males, 11 females). *G*, serum concentrations of GDF15 at the end of the experiment. n = 23 (12 males, 11 females). Box plots show median values, *top* and *bottom* hinges refer to the first and third quartiles (25th and 75th percentiles), and the ends of the whiskers mark the smallest and largest values. Bar charts show mean values ± SD. Data in each graph were analyzed by a two-way ANOVA (genotype x drug). If a statistical interaction occurred, groups not sharing a common letter indicated a statistically significant difference between groups after *post hoc* pairwise comparisons were conducted with a Tukey correction for multiple comparisons. Significance threshold = *p* ≤ 0.05.

We next sought to determine if the blunted ISR response to PEG-asparaginase in *Atg7*^Δ/Δ^ mice was liver autonomous. To accomplish this, we injected mice with an AAV-TBG-iCre to induce liver-specific deletion of *Atg7* (ls-*Atg7*KO) or an AAV-TBG-GFP control (*Atg7*^F/F^) then treated these mice with a single injection of PEG-asparaginase or PBS vehicle 3 weeks later (Fig. 4*A*). There were no differences in starting body weight, lean mass, or fat mass between any groups of mice (Fig. S7, *A*–*C*). Effective and specific knockdown of the ATG7 protein in the liver but not the kidney was confirmed by immunoblot (Fig. S7, *D*–*F*). MRIs were administered to all mice before exposure to PEG-asparaginase and then 5 days later, before euthanasia. Similar to *Atg7*^Δ/Δ^ mice, ls-*Atg7*KO mice exposed to PEG-asparaginase lost less weight as compared to *Atg7*^F/F^ mice (Fig. 4*B*). However, ls-*Atg7*KO mice were not protected from a loss in fat mass (Fig. 4*C*). Instead, ls-*Atg7*KO mice were only protected from a reduction in lean mass (Fig. 4*D*). Serum concentrations of FGF21 mirrored the pattern observed in the earlier *Atg7*^+/+^ *versus Atg7*^Δ/Δ^ study; serum concentrations of FGF21 were significantly increased in all PEG-asparaginase treated mice, but ls-*Atg7*KO mice were blunted as compared to *Atg7*^F/F^ mice (Fig. 4*E*). PEG-asparaginase decreased food intake in both groups of mice (Fig. 4*F*) and elevated serum concentrations of GDF15 similarly in ls-*Atg7*KO and *Atg7*^F/F^ mice (Fig. 4*G*). These findings show that intact *Atg7* in the liver corresponds with maximal increases in serum FGF21 during PEG-asparaginase exposure. In contrast, increased GDF15 and decreased food intake in response to PEG-asparaginase are not modulated by hepatic *Atg7* status.

### Liver-specific loss of *Atg7* suppresses transcriptional execution of the integrated stress response in the liver

**Figure 5:**
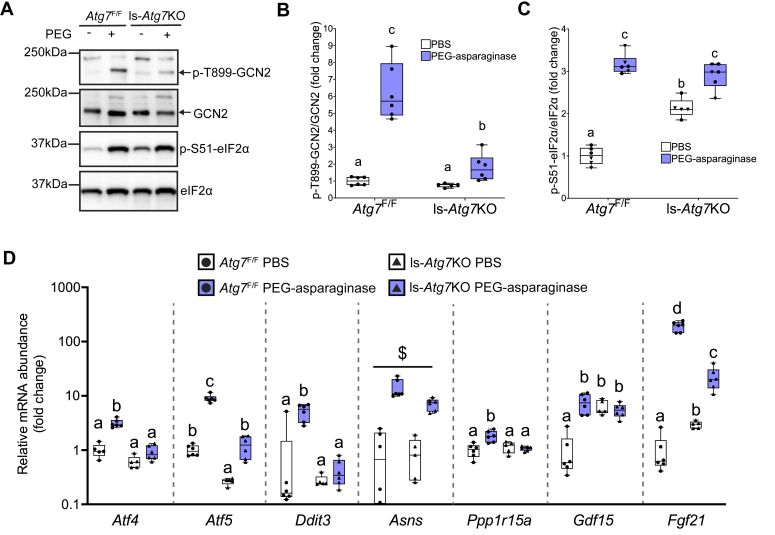
**Hepatic *Atg7* modulates the ISR in the liver.***A*, Representative immunoblots of p-T899-GCN2/total GCN2 ratio and p-S51-eIF2α/total eIF2α. *B*, quantification of p-T899-GCN2/total GCN2 ratio expressed as fold change of PBS-treated *Atg7*^+/+^ mice. n = 23 (12 males, 11 females). *C*, quantification of p-S51-eIF2α/total eIF2α expressed as fold change of PBS-treated *Atg7*^+/+^ mice. n = 23 (12 males, 11 females). *D*, relative mRNA abundance by qPCR analysis of ISR target genes *Atf4*, *Atf5*, *Ddit3*, *Asns*, *Ppp1r15a*, *Gdf15* and *Fgf21*. n = 23 (12 males, 11 females). Box plots show median values, *top* and *bottom* hinges refer to the first and third quartiles (25th and 75th percentiles), and the ends of the whiskers mark the smallest and largest values. Panels *B*–*C*: • = females. ▼ = males. Data in each graph were analyzed by a two-way ANOVA (genotype x drug). $ represents the significant main effect of the drug. If a statistical interaction occurred, groups not sharing a common letter indicated a statistically significant difference between groups after *post hoc* pairwise comparisons were conducted with a Tukey correction for multiple comparisons. Significance threshold = *p* ≤ 0.05.

To determine if hepatic *Atg7* modulates the broader ISR basally or upon PEG-asparaginase exposure, we measured phosphorylation of GCN2 and eIF2α as well as mRNA abundance of ISR target genes in the liver. Similar to *Atg7*^Δ/Δ^ mice, the livers of ls-*Atg7*KO mice showed lower GCN2 activation and hyperphosphorylation of eIF2α as compared to *Atg7*^F/F^ mice (Fig. 5, *A*–*C*). Additionally, hepatic loss of *Atg7* suppressed the PEG-asparaginase-induced increase in the ISR target genes *Atf4*, *Atf5*, *Ddit3*, *Ppp1r15a*, *Gdf15*, and *Fgf21* but not *Asns* (Fig. 5*D*). In summary, loss of hepatic *Atg7* was sufficient to induce hyperphosphorylation of eIF2α and suppress the transcriptional execution of the ISR in the liver in response to PEG-asparaginase.

### Loss of hepatic *Atg7* prevents PEG-asparaginase-induced hepatic steatosis

**Figure 6:**
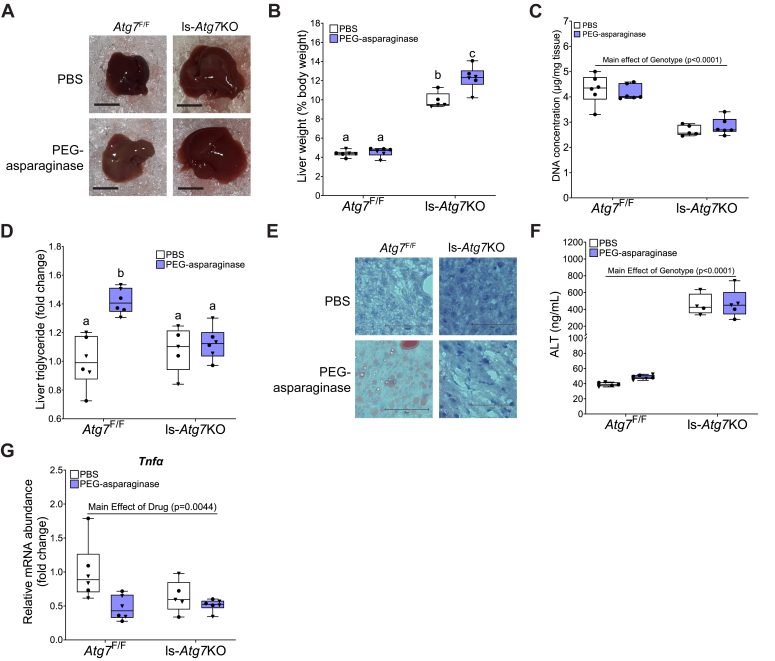
**Mice with a liver-specific knockdown of *Atg7* are protected from PEG-asparaginase-induced hepatic steatosis.***A*, whole livers of *Atg7*^F/F^ and ls-*Atg7*KO mice exposed to PEG-asparaginase (1.5 IU/g) or an equivolume of PBS as the vehicle. Scale bars are 1 cm. *B*, liver weight expressed as a percentage of body weight. n = 23 (12 males, 11 females). *C*, hepatic DNA concentration. n = 23 (12 males, 11 females). *D*, hepatic triglyceride abundance, normalized to DNA content, expressed as fold change of *Atg7*^F/F^ mice exposed to PBS. n = 23 (12 males, 11 females). *E*, oil Red O and hematoxylin staining on representative liver sections of *Atg7*^F/F^ and ls-*Atg7*KO mice exposed to PEG-asparaginase or PBS control. Scale bar is 75 μM. *F*, serum concentrations of ALT at the end of the experiment. n = 20 (11 males, 9 females). *G*, hepatic mRNA abundance of *Tnfα* analyzed by qPCR. n = 23 (12 males, 11 females). Box plots show median values, *top* and *bottom* hinges refer to the first and third quartiles (25th and 75th percentiles), and the ends of the whiskers mark the smallest and largest values. • = females. ▼ = males. Data in each graph were analyzed by a two-way ANOVA (genotype x drug). If a statistical interaction occurred, groups not sharing a common letter indicated a statistically significant difference between groups after *post hoc* pairwise comparisons were conducted with a Tukey correction for multiple comparisons. Significance threshold = *p* ≤ 0.05.

We next sought to determine how the liver-specific loss of autophagy affects hepatic steatosis, hepatotoxicity, and inflammation basally and in response to PEG-asparaginase. Livers of *Atg7*^F/F^ mice after exposure to PEG-asparaginase were pale in appearance upon dissection (Fig. 6*A*). In contrast, the livers of PEG-asparaginase-treated ls-*Atg7*KO mice were not pale in appearance (Fig. 6*A*). Loss of hepatic *Atg7* increased liver weight (Fig. 6*B*). PEG-asparaginase also increased liver weight relative to body weight in ls-*Atg7*KO mice but not *Atg7*^F/F^ mice (Fig. 6*B*). Overall, the concentration of DNA in the livers of ls-*Atg7*KO mice was lower than that of *Atg7*^F/F^ mice while PEG-asparaginase exposure had no effect (Fig. 6*C*). Triglycerides were significantly increased in the livers of *Atg7*^F/F^ mice but not in ls-*Atg7*KO mice (Fig. 6*D*). Oil Red O staining on liver sections confirmed PEG-asparaginase-induced increased lipids only in *Atg7*^F/F^ mice (Fig. 6*E*). Similar to results in *Atg7*^Δ/Δ^ mice, knockdown of hepatic *Atg7* significantly elevated serum ALT concentrations but PEG-asparaginase had no effect (Fig. 6*F*). Also similar to the earlier *Atg7*^+/+^ *versus Atg7*^Δ/Δ^ study, PEG-asparaginase decreased hepatic *Tnfα* mRNA, independent of genotype (Fig. 6*G*). Overall, these results indicate that autophagy participates in the development of PEG-asparaginase-induced hepatic steatosis.

## Discussion

**Figure 7:**
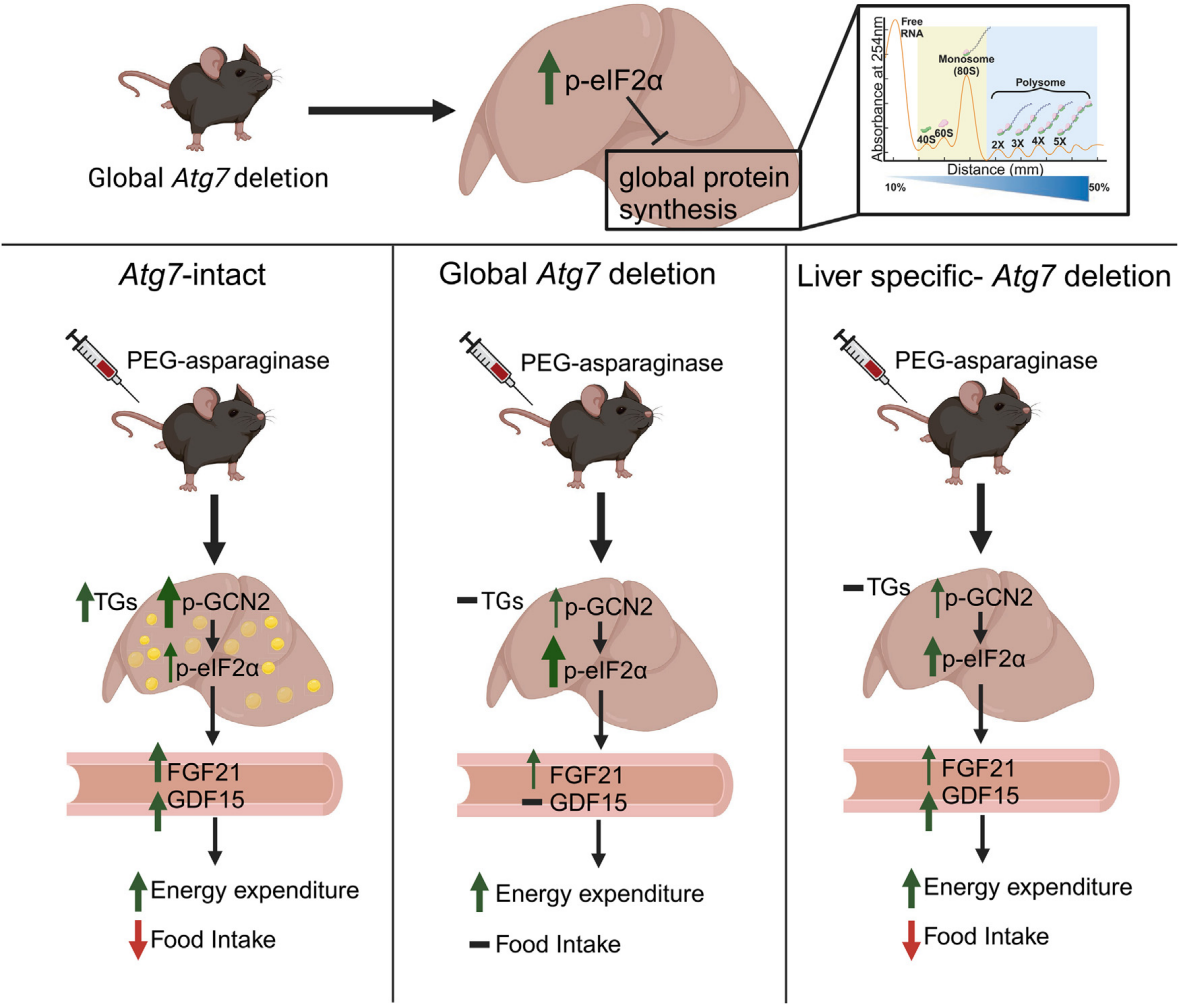
**Graphical model of *Atg7*-dependent outcomes during exposure to PEG-asparaginase.***Atg7* modulates hepatic triglyceride content and circulating stress-response hormones.

The ISR is necessary to protect mice from hepatic steatosis, hepatotoxicity, and eventual death due to asparaginase exposure (10, 11, 19, 39). Upregulation of autophagy is a well-established process initiated by activation of the ISR (20, 25, 39, 40, 41, 42). Loss of ISR function by various means, including knocking down *Gcn2*, *Atf4*, and *Ddit3*, prevents the transcription of many autophagy genes during leucine deprivation (20). Data on the role of autophagy during the depletion of asparagine by asparaginase is limited, but prior work in leukemic lymphoblasts shows that asparaginase upregulates autophagy and that inhibition of autophagy decreases cell survival (25). The present study aimed to examine the role of autophagy during exposure to the chemotherapeutic asparaginase by deleting a core autophagy gene, *Atg7*, in mice. We find the global loss of *Atg7* prevents weight loss induced by PEG-asparaginase, decreases global translation basally, and blocks the PEG-asparaginase-induced transcriptional execution of the ISR in the liver (Fig. 7). Loss of *Atg7* in the liver also protects mice from PEG-asparaginase-induced weight loss, hepatic steatosis, and blunts the transcriptional execution of the ISR in the liver. Energy expenditure and food intake in both models are consistent with changes in circulating FGF21 and GDF15 levels, respectively. Maximal FGF21 expression is dependent on hepatic *Atg7*, but GDF15 expression is not. These findings provide novel insight into the function of autophagy in response to PEG-asparaginase.

Our findings show that not only is autophagy regulated by the ISR, but also provides feedback to regulate the ISR itself. We hypothesized that ISR activation would be higher in *Atg7*^Δ/Δ^ mice, but our results do not support this. The cause of lower GCN2 activation in the livers of *Atg7*-deficient mice during exposure to PEG-asparaginase remains elusive. Deacetylated tRNAs, ribosomal stalling, and ribosome collisions can activate GCN2 (43, 44). How basal or stress-induced autophagy may regulate either of these mechanisms to sustain GCN2 activation is yet to be determined. Lower GCN2 activation, with no activation of PERK, leads us to speculate that eIF2α is hyperphosphorylated in *Atg7*-deficient mice due to decreased eIF2α phosphatase activity. Our data that shows lower mRNA expression of *Ppp1r15a*, the gene that encodes for the eIF2α phosphatase, GADD34, supports this hypothesis. These data align with previous reports of increased eIF2α phosphorylation in GADD34-deficient mice (45, 46). An important finding in our study is that the liver-specific loss of *Atg7* is sufficient to decrease GCN2 activation and lead to elevated eIF2α phosphorylation in the liver. This indicates that *Atg7* regulates the ISR in local tissue and is not mediated by changes in inter-organ metabolism that may occur when *Atg7* is reduced globally. An alternate explanation is that a reduction in the ISR is a consequence of extreme cell death. However, PEG-asparaginase did not further reduce DNA content or increase circulating ALT, a measure of liver toxicity, in either of the *Atg7*-deficient models (Figs. 3*C* and 6*C*). Additional efforts to examine apoptosis also showed no increases in caspase-3 cleavage by PEG-asparaginase (Fig. S9). Additional experiments are required to resolve the influence of *Atg7* knockdown on the ISR.

We examined how hyperphosphorylation of eIF2α in the livers of *Atg7*-deficient mice affects rates of global translation by conducting polysome profile analyses. The role of autophagy in regulating protein synthesis is highly context-dependent. Protein synthesis rates of mouse embryonic fibroblasts in the fed and fasted state are unaffected by the presence of autophagy (47). However, in our experiments, we found global translation is lower in the livers of autophagy-deficient mice in the unstressed state. Thus, autophagy is necessary to maintain protein synthesis *in vivo*. An inability to restore cytosolic amino acid levels in the livers of *Atg7*-deficient mice is not likely to cause reduced protein synthesis, as the liver amino acid pool in *Atg7*^Δ/Δ^ mice is not different relative to wild-type mice (23). Interestingly, hepatic global translation during exposure to PEG-asparaginase is not different between autophagy-intact and autophagy-deficient mice. We propose the potency of PEG-asparaginase to reduce global protein synthesis supersedes the role of autophagy to maintain translation. Overall, we find that *Atg7* is necessary to sustain protein synthesis in the liver.

A hallmark of the ISR is the preferential translation of stress response transcription factors, such as ATF4, with a concomitant decrease in global translation rates. Previous work by our lab shows that *Atf4* mRNA abundance shifts from the light fraction to the heavy fraction of polysomes in the livers of mice exposed to asparaginase compared to control (48). In the current study, mRNA abundance of ATF4 target genes is lower in the livers of *Atg7*-deficient mice. A possible explanation for this is the decreased synthesis of ATF4 in response to PEG-asparaginase. Goldsmith and colleagues previously showed that accumulation of p62 aggregates during autophagy deficiency sequesters the eukaryotic initiation factor 4E (eIF4E), preventing translation of a subset of mRNA transcripts which includes *Atf4* (47). Danieli *et al.*, recently showed that in addition to eIF4E, eIF2 is also found in p62 condensates of Hap1 cells. Indeed, autophagy-deficient mice in our study express high levels of p62 in the liver. Future experiments examining transcriptomic analysis across polysome fractions may be useful in discerning the role of ATG7 in guiding gene-specific translation during PEG-asparaginase exposure. In sum, these findings indicate that autophagy is required for the transcriptional execution of the ISR in the liver in response to PEG-asparaginase.

Importantly, we find differences in circulating protein levels of the ATF4 target genes, GDF15 and FGF21. GDF15 is an anorexic hormone regulated by the ISR during nutrient stress *via* the GDNF family receptor α-like (GFRAL) receptor in the brain (33, 34, 49, 50, 51). This is the first study, to our knowledge, to show PEG-asparaginase increases serum levels of GDF15. Previous work by our lab shows that asparaginase produces an anorexic effect in mice (9). We propose that GDF15 is responsible for producing this anorexic effect, similar to dietary essential amino acid insufficiency (34). In support of this hypothesis, PEG-asparaginase does not increase serum concentrations of GDF15 above baseline nor does it decrease food intake in *Atg7*-deficient mice. Additionally, mice with a liver-specific deletion of *Atg7* have elevated GDF15 and decreased food intake. Our conclusions from these data are two-fold: GDF15 in the serum corresponds with changes in food intake in response to PEG-asparaginase; hepatic *Atg7* is not necessary to elevate GDF15 under these conditions. Other tissues, including adipose tissue, are substantial contributors to serum GDF15 levels during ISR activation (50). An alternative explanation for *Atg7*-driven changes in food intake is the regulation of appetite locally by *Atg7* in the brain. One report shows that loss of *Atg7* in the hypothalamus decreases food intake during post-starvation refeeding (52). Given that autophagy-deficient mice in our study display a greater food intake relative to *Atg7*-intact mice in response to PEG-asparaginase, a lack of autophagy in the hypothalamus is unlikely to produce the phenotype herein observed. Loss of *Atg7* also decreases the expression of FGF21. FGF21 is a hormone that is elevated under various forms of stress, including amino acid insufficiencies, that increases energy expenditure (18, 53, 54, 55). Unlike GDF15, maximal increases in FGF21 in response to PEG-asparaginase are dependent on *Atg7* in the liver. PEG-asparaginase increases energy expenditure in both groups of mice, which may be FGF21-driven, implying that lower levels of FGF21 in the serum are sufficient to increase energy expenditure. Our finding that FGF21 was also lower in the ls-*Atg7*KO mice indicates that, unlike GDF15, FGF21 is primarily liver-derived and that autophagy is necessary to maximally increase FGF21 during PEG-asparaginase exposure.

Given that *Atg7*-deficient mice and humans with genetic polymorphisms in the *Atg7* gene develop fatty liver disease (56), we hypothesized the autophagic degradation of hepatic lipids may mitigate PEG-asparaginase-induced hepatic steatosis. Contrary to our hypothesis, we found that *Atg7* is a contributor to PEG-asparaginase-induced hepatic steatosis. Our data in this study are aligned with studies conducted by Kwenton *et al.*, and Ma *et al.*, which show that loss of autophagy in the liver protects mice from fasting-induced hepatic steatosis (57, 58). Although the mechanism behind this finding is unclear, *Atg7* is also implicated in the formation of lipid droplet membranes (59). Data from our lab and others supports a model in which asparaginase activates adipose tissue lipolysis *via* FGF21 (60), leading to an accumulation of free fatty acids in the liver (10, 61). We speculate that the deficiency of *Atg7* prevents the storage of adipose-derived fatty acids in lipid droplets, resulting in resistance to hepatic steatosis.

We also found that mice with a whole-body deletion of *Atg7* are moderately protected from PEG-asparaginase-induced weight loss. The finding that food intake, but not energy expenditure, differs between *Atg7*-intact and *Atg7*-deficient mice, implies that early loss of *Atg7* protects mice from weight loss through a derepression of food intake in response to PEG-asparaginase. These data are consistent with results from previous experiments performed by our lab that show a decrease in food intake contributes to, but does not wholly account for, asparaginase-induced weight loss (18). Interestingly, mice with a liver-specific loss of *Atg7* are also protected from PEG-asparaginase-induced weight loss but exhibited normal increases in GDF15 and decreases in food intake. Blunted weight loss in ls-*Atg7*KO mice could be attributed to a blunted increase in energy expenditure, although energy expenditure was not measured in these mice. Our data supports an ATG7-GDF15 axis underlying the PEG-asparaginase-induced decrease in food intake. Future studies exploring PEG-asparaginase exposure in mice deficient in *Gdf15* and/or *Fgf21* are warranted. Given that *Gdf15* and *Fgf21* are both implicated in physiological outcomes associated with cancer treatment, these results provide an important foundational understanding of the biological pathways that initiate their expression.

## Experimental procedures

### Animal care

All experiments were approved by the Rutgers University Institutional Animal Care and Use Committee in compliance with the NIH guide for the care and use of laboratory animals and in accordance with the ARRIVE 2.0 guidelines (62). Mice were housed in transparent plastic shoebox cages with soft bedding and environmental enrichment and provided free access to purified water and commercial rodent chow (5058- PicoLab Mouse Diet 20). All mice were maintained in a temperature-controlled (23 °C) facility with a 12h light/dark cycle.

### Experiment 1

Twenty-two wild-type C57BL6/J mice (11 male, 11 female) aged 15 to 30 weeks were administered an intraperitoneal (IP) injection of either polyethylene glycol (PEG)ylated asparaginase (PEG-asparaginase) (Oncaspar) at 1.5 international units per gram (IU/g) body weight, 3.0 IU/g body weight, or an equivalent volume of phosphate-buffered saline (PBS) as vehicle at 9:00 to 10:00 on Day 0. Mice were single-housed starting 1 day before injection of the drug or excipient and monitored throughout the study for signs of distress and morbidity until the end of the experiment. All mice were killed by decapitation 5 days later at 15:00 to 16:00 in the afternoon. Organs were rapidly dissected on ice, frozen in liquid nitrogen, and stored at −80 °C until future analyses.

### Experiment 2

Twenty-five (6 male, 19 female) Ubc*-Cre*ERT2^/+^*Atg7*^+/+^ and twenty-three (7 male, 16 female) *Ubc-Cre*ERT2^/+^*Atg7*^F/F^ mice aged 8 to 10 weeks were first administered 4 daily injections of Tamoxifen resulting in *Atg7*-intact (*Atg7*^+/+^) and *Atg7*-deficient (*Atg7*^Δ/Δ^) mice, respectively. A description of the creation of this genetic mouse model is published (23). Three weeks post-injection with tamoxifen (Day 0), body composition was analyzed by MRI (EchoMRI), and mice were administered an (IP) injection of either PEG-asparaginase (1.5 IU/g body weight) or an equivalent volume of PBS. Mice were single-housed starting 1 day before injection of the drug or excipient and monitored throughout the study for signs of distress and morbidity until the end of the experiment. A subset of 13 females (7 *Atg7*^Δ/Δ^, 6 *Atg7*^+/+^) was housed on wire bottom inserts during the experiment to allow for accurate measurement of food intake. A separate subset of 12 female *Atg7*^+/+^ and *Atg7*^Δ/Δ^ mice were placed in the OxyMax Comprehensive Lab Animal Monitoring System (CLAMS) (Columbus Instruments) to measure energy expenditure. To sufficiently acclimate mice to a new environment, mice were placed in the CLAMS cages for 3 days before the start of the study. On Day 0, mice were removed from the cages individually between interval measurements and administered an IP injection of PEG-asparaginase (1.5 IU/g body weight) or an equal volume of PBS and then returned to their respective cages. Mice were removed from the CLAMS on Day 5 and killed by decapitation at 15:00. To calculate energy expenditure before and after exposure to PEG-asparaginase or PBS, cumulative energy expenditure for 24 h before injection and for 24 h before euthanasia was normalized to body weight at their respective time points. All mice in Experiment 2 were killed by decapitation 5 days post-administration of PEG-asparaginase or PBS at 15:00 to 16:00 in the afternoon. Organs were rapidly dissected on ice, frozen in liquid nitrogen, and stored at −80 °C until future analyses.

### Experiment 3

Twenty-three 8 to 10-week-old *Atg7*^F/F^ mice were used to generate liver-specific *Atg7* knockdown mice (ls-*Atg7*KO) and *Atg7*^F/F^ control mice, respectively, as previously described (24). Liver-specific *Atg7* deletion was first achieved *via* injection with an adeno-associated virus (AAV)-thyroxine-binding globulin (TBG) promoter-Cre recombinase vector (AAV-TBG-iCre, Vector Biolabs) to *Atg7*^F/F^ mice. As a control, AAV-TBG promoter-GFP vector (AAV-TBG-GFP, Vector Biolabs) was injected into *Atg7*^F/F^ mice. 1.4 × 10∧11 genome copy of either AAV-TBG-iCre or AAV-TBG-GFP vectors in 100 μl PBS was injected into the tail vein. Three weeks post-injection with the respective AAV (Day 0), body composition was analyzed by MRI (EchoMRI) and mice were administered an intraperitoneal (IP) injection of either PEG-asparaginase (1.5 IU/g body weight) or an equivalent volume of PBS. Mice were single-housed starting 1 day before injection of drug or excipient and monitored throughout the study for signs of distress and morbidity until the end of the experiment. All mice in Experiment 3 were killed by decapitation 5 days post-administration of PEG-asparaginase or PBS at 15:00 to 16:00 in the afternoon. Organs were rapidly dissected on ice, frozen in liquid nitrogen, and stored at −80 °C until future analyses.

### PEG-asparaginase enzymatic activity

The enzymatic activity of the PEG-asparaginase solution was determined by the Nesslerization technique as previously described (8). Prior to administration, PEG-asparaginase activity was measured by ammonia production over time in a solution of asparagine and compared to an ammonia standard curve. Results were represented in international units. One IU represents the amount of enzyme that produces 1 μmol of ammonia per minute.

### Immunoblot analyses

Total protein was isolated from approximately 25 mg of frozen, crushed liver tissue using a radioimmunoprecipitation assay lysis buffer solution consisting of 25 mM HEPES, 2 mM EDTA (Invitrogen), 10 mM dithiothreitol, 50 mM sodium fluoride (Alfa Aesar), 50 mM β-glycerophosphate pentahydrate (Alfa Aesar), 3 mM benzamidine, 1 mM sodium orthovanadate, 0.5% (w/v) sodium deoxycholate, 1% (w/v) sodium dodecyl sulfate (Fisher), 5 nM microcystin, and 1x protease inhibitor cocktail (P8340, Millipore-Sigma. All reagents were purchased from Millipore-Sigma unless otherwise stated. Samples were homogenized in a 1:30 (w:v) ratio in 1.5 ml microcentrifuge tubes with a motorized pestle on ice. Lysates were centrifuged at 10,000*g* for 10 min at 4 °C. The supernatant was removed and mixed with an equal amount (v:v) of 2x sample buffer solution containing 20% (v/v glycerol), 60 mM Tris (pH 6.8), 2% (w/v) SDS, 5% (v/v) β-mercaptoethanol, and 0.01% (w/v) bromophenol blue. Samples were subsequently heated at 95 °C for 3 min and stored at −80 °C. Gel electrophoresis was performed by loading equal volumes of samples onto SDS-polyacrylamide gels. Proteins were transferred to PVDF membranes that were then blocked in 5% (w/v) non-fat milk for 1 h at room temperature. Membranes were washed and incubated with a primary antibody overnight at 4 °C on a tabletop rocker. The following day, membranes were washed and incubated in a secondary antibody for 1 h at room temperature in a 5% (w/v) non-fat milk solution. Membranes were then incubated for 1 min in enhanced chemiluminescence solution (RPN2235, Cytvia Amersham ECL Select Western Blotting Detection Agent, Cytvia) to image the targeted proteins (FluorChem M; ProteinSimple). Densitometry was performed with ImageJ software (Fiji). Values were normalized to total protein (Coomassie stain), except for phosphorylated proteins which were normalized to their respective total forms and expressed as fold change of *Atg7*-intact control mice. All antibodies were purchased from commercial vendors and details are listed in Table S1.

### Polysome profiles

Polysome profiling analyses from sucrose gradients were performed as described previously (32, 48, 63), but with some modifications as detailed below. Approximately 75 mg of frozen liver tissue was homogenized with plastic polypropylene pestles (12-141-364, Thermo Fisher Scientific) in a 1.5 ml microcentrifuge tube using a 1:10 (w:v) ratio of lysis buffer solution of 25 mM HEPES (pH 7.5), 100 mM KCl, 10 mM MgCl_2_, 0.3% (w/v) sodium deoxycholate, 1% (v/v) Triton X-100, 1 mM DTT, 1x protease inhibitor cocktail (P8340, Millipore-Sigma), and 1200 U SUPERase-In RNase inhibitor (AM2696, Thermo Fisher Scientific). Lysates were incubated on ice for 10 min and subjected to centrifugation at 10,000*g* for 10 min at 4 °C. The cleared supernatants were collected, and 0.5 ml was loaded on top of 10% - 50% (w/v) linear sucrose gradients containing 25 mM HEPES (pH 7.5), 10 mM MgCl_2_ and 100 mM that were prepared using a Gradient Master (BioComp Instruments, Fredericton, New Brunswick, Canada). Sucrose gradients with the tissue supernatants on top were equilibrated using 10% (w/v) sucrose solution and subjected to centrifugation at 100,000*g* for 3.5 h at 4 °C using a JS-24.38 swing bucket rotor (Beckman Coulter) in an Avanti J-301centrifuge (Beckman Coulter). After centrifugation, the sucrose gradients were fractionated into 1 ml aliquots using a piston gradient fractionator (BioComp Instruments, Fredericton) that continuously recorded the absorbance at 254 nm. Global protein synthesis was estimated by calculating the ratio of the combined area under the curve (AUC) of the polysome (disome and greater) to the combined AUC of the 40S, 60S, and monosome fractions.

### RNA isolation and real-time quantitative PCR

Total RNA was isolated from approximately 15 mg of frozen, crushed liver tissue in 750 μl TRI Reagent RT (TR 119, Molecular Research Center, Inc.). In brief, samples were homogenized with a handheld motorized pestle, incubated on ice for 5 min, mixed with 100 μl of BAN, vortexed, and centrifuged at 12,000*g* for 15 min at 4 °C. The supernatant was removed, mixed with isopropanol, and centrifuged at 12,000*g* for 5 min at 4 °C. Pellets were washed three times with 70% (v/v) ethanol, treated with DNase, and diluted in nuclease-free water. RNA quality and concentration were determined by gel electrophoresis and spectrophotometry on a NanoDrop, respectively. RNA was reverse transcribed to cDNA (High-Capacity cDNA Reverse Transcription Kit, Applied Biosystems), and changes in relative transcript abundance were analyzed by qPCR (PowerUp SYBR Green Master Mix, Applied Biosystems). Primer sequences are listed in Table S2.

### Serum protein concentrations

Serum ALT concentrations were measured using a Mouse ELISA kit (Abcam, ab282882) per the manufacturer’s instructions. Samples were diluted 1:100 prior to analysis. Serum FGF21 concentrations were measured using an FGF21 Mouse/Rat ELISA kit (BioVendor, RD 291108200R) per the manufacturer’s instructions. Samples were diluted 1:10 prior to analysis. Serum GDF15 concentrations were measured using the Mouse/Rat GDF-15 Quantikine ELISA kit (R&D Systems, MGD150) following the manufacturer’s instructions. Samples were diluted 1:50 prior to analysis. All resulting absorbances were measured using a 96-well microplate reader (SpectraMax M2; Molecular Devices) and concentrations were calculated using the 4PL method in GraphPad Prism.

### Hepatic triglycerides

Approximately 40 mg of frozen, crushed liver tissue was homogenized in 10% (v/v) NP-40 (Amresco, J619) using a handheld, motorized pestle. Samples were further processed per the manufacturer’s instructions (Abcam, Triglyceride Assay Kit- Quantification, ab65336). Total DNA concentration was measured by using the Zymo *Quick*-DNA Miniprep Kit (D3024). All triglyceride values were normalized to DNA concentration.

### Oil Red O

Frozen liver sections of 5 μm were cut and stained with Oil Red O and hematoxylin per the manufacturer’s instructions (Abcam ab150678).

### Statistical analyses

Data were tested for normality using the Shapiro-Wilk normality test. Data that were not normally distributed were log_10_ transformed to achieve normality prior to statistical analyses. Unless otherwise stated, data were analyzed using a two-factor ANOVA to assess the main effects of independent variables: genotype and drug. A Tukey’s test for multiple comparisons was performed if a statistically significant interaction occurred. The significance level was set to *p* ≤ 0.05 for all tests. GraphPad Prism was used to conduct statistics and generate graphs. Box plots show median values, top and bottom hinges refer to the first and third quartiles (25th and 75th percentiles), and the ends of the whiskers mark the smallest and largest values. Bar graphs show means ± SD.

## Data availability

All data described are contained within this article or the Supporting Information.

## Supporting information

This article contains supporting information.

## Conflict of interest

The authors declare that they have no conflicts of interest with the contents of this article.
