# Supplementary material for: Autophagy-related 7 (ATG7) regulates food intake and liver health during asparaginase exposure
Source: J Biol Chem. 2025 Jan 10;301(2):108171. doi: 10.1016/j.jbc.2025.108171 (PMC11850126; doi:10.1016/j.jbc.2025.108171)
Supplement: Supporting information [file mmc1.pdf]

Autophagy related 7 (ATG7) regulates food intake and liver health during asparaginase exposure.

Brian A. Zalma, Maria Ibrahim, Flavio C. Rodriguez-Polanco, Chintan T. Bhavsar, Esther M. Rodriguez, Eduardo Cararo-Lopes, Saad A. Farooq, Jordan L. Levy, Ronald C. Wek, Eileen White, and Tracy G. Anthony

Supplemental Figure 1: Administration of PEG-asparaginase (Oncaspar®) at 1.5 IU/g body weight or 3.0 IU/g body weight elicits similar weight loss and eIF2α phosphorylation in the liver.

Supplemental Figure 2: Starting body weight and composition of *Atg7*$^{+/+}$ and *Atg7*$^{\Delta/\Delta}$ mice.

Supplemental Figure 3: PEG-asparaginase induces loss of adipose tissue in epididymal and inguinal fat pads.

Supplemental Figure 4: Energy expenditure, respiratory exchange ratio, activity, and cumulative food intake in the CLAMS.

Supplemental Figure 5: Loss of *Atg7* does not activate PERK in the liver during PEG-asparaginase exposure.

Supplemental Figure 6: Polysome profiling in the livers of *Atg7*$^{+/+}$ and *Atg7*$^{\Delta/\Delta}$ mice

Supplemental Figure 7: Starting body composition of ls-*Atg7*KO and *Atg7*$^{F/F}$ mice and validation of liver-specific *Atg7* knockdown.

Supplemental Figure 8: Loss of hepatic *Atg7* decreases fat pad weight.

Supplemental Figure 9: Knockdown of ATG7 does not induce cleavage of caspase-3 in the liver.

Supplemental Table 1: Antibodies.

Supplemental Table 2: qPCR primers.

Supplemental Figure 1

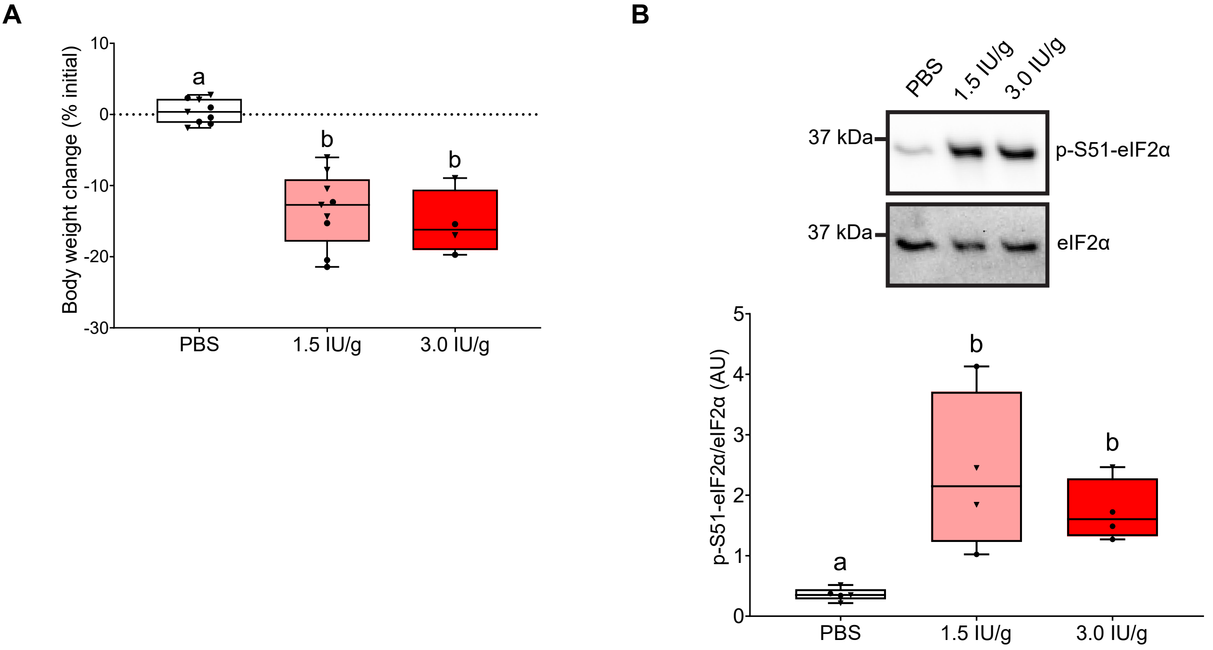

Supplemental Figure 1: Administration of PEG-asparaginase (Oncaspar®) at 1.5 IU/g body weight or 3.0 IU/g body weight elicits similar weight loss and eIF2α phosphorylation in the liver.

(A) Body weight change in mice exposed to PBS, 1.5 IU/g body weight, or 3.0 IU/g body weight of PEG-asparaginase n= 22 (11 males, 11 females). (B) Representative immunoblot of eIF2α phosphorylation in the liver and quantification**.** n= 13 (5 males, 8 females). Box plots show median values, top and bottom hinges refer to the first and third quartiles (25th and 75th percentiles), and the ends of the whiskers mark the smallest and largest values. ●= females. ▼= males. Data in each graph analyzed by a one-way ANOVA. Groups not sharing a common letter indicated a statistically significant difference between groups after Dunnett's T3 multiple comparisons post-hoc test. Significance threshold = $p \leq 0.05$.

Supplemental Figure 2

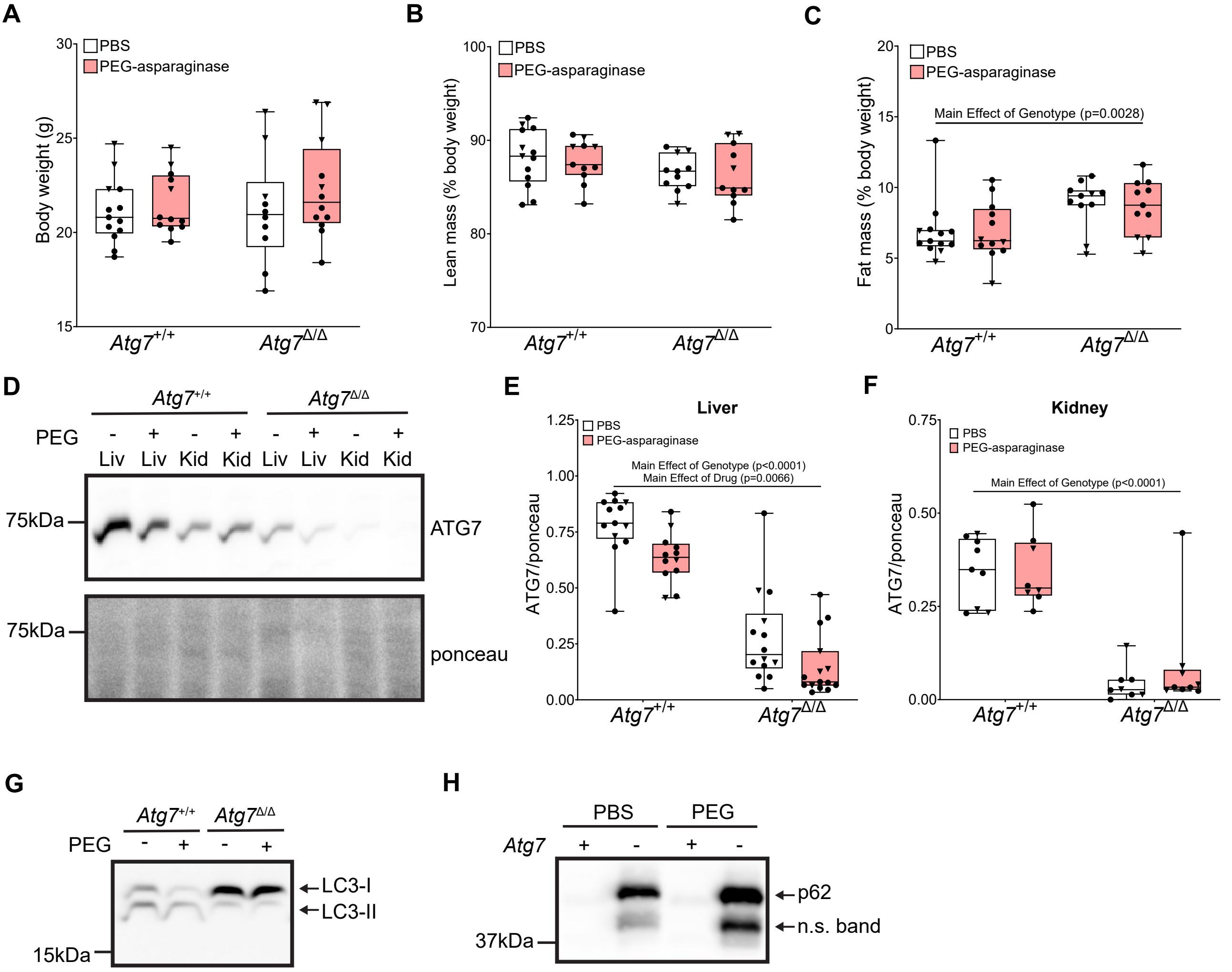

Supplemental Figure 2: Starting body weight and composition of *Atg7*$^{+/+}$ and *Atg7*$^{\Delta/\Delta}$ mice.

(A) Body weight at IP administration of PEG-asparaginase or PBS. n= 48 total (3-4 males, 6-7 females, per group). (B) Body lean mass, expressed as percent of initial body weight, at IP administration of PEG-asparaginase or PBS. 47 total (3-4 males, 6-7 females, per group). (C) Body fat mass, expressed as percent of initial body weight, at IP administration of PEG-asparaginase or PBS. 47 total (3-4 males, 6-7 females, per group). (D) Representative immunoblot of ATG7 protein in the liver and kidney. (E) Quantification of ATG7 protein in the liver normalized to total protein (ponceau). (F) Quantification of ATG7 protein in the kidney normalized to total protein (ponceau). (G) Immunoblot of LC3 protein in the liver. (H) Immunoblot of p62 protein in the liver. Box plots show median values, top and bottom hinges refer to the first and third quartiles (25th and 75th percentiles), and the ends of the whiskers mark the smallest and largest values. ●= females. ▼ = males. Data in each graph analyzed by a two-way ANOVA (genotype x drug).

Supplemental Figure 3

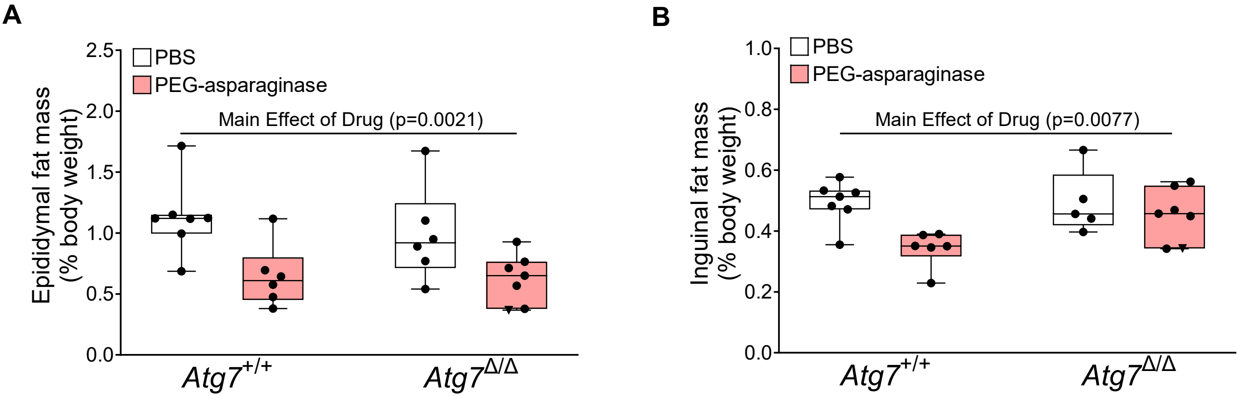

Supplemental Figure 3: PEG-asparaginase induces loss of adipose tissue in epididymal and inguinal fat pads.

(A) Epididymal fat pad weight expressed as percent of body weight. n= 26 (1 male, 25 females). (B) Inguinal fat pad weight expressed as percent of body weight. n= 26 (1 male, 25 females). Box plots show median values, top and bottom hinges refer to the first and third quartiles (25th and 75th percentiles), and the ends of the whiskers mark the smallest and largest values. ● = females. ▼ = males. Data in each graph analyzed by a two-way ANOVA (genotype x drug).

Supplemental Figure 4

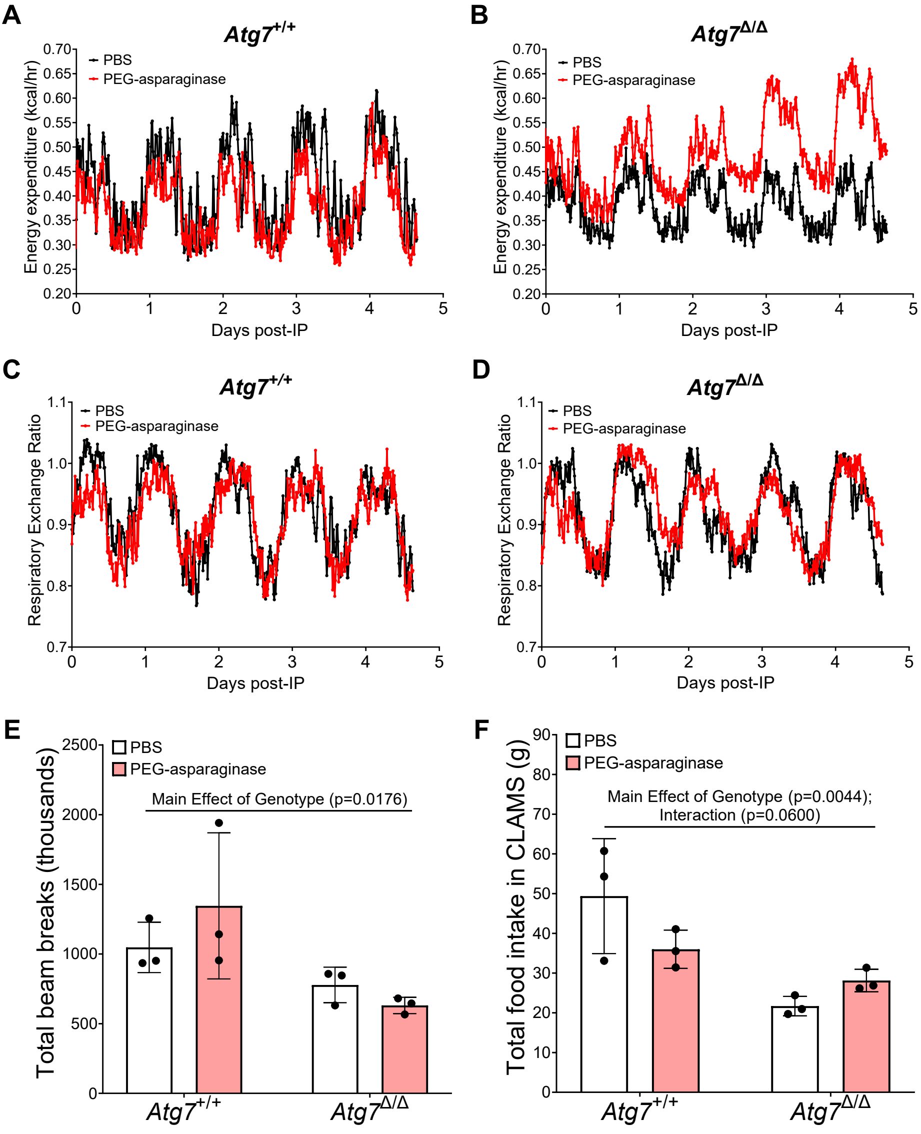

Supplemental Figure 4: Energy expenditure, respiratory exchange ratio, activity, and cumulative food intake in the CLAMS.

(A) Energy expenditure of *Atg7*$^{+/+}$ mice exposed to PEG-asparaginase or PBS for 5 days. n= 12 (12 females). (B) Energy expenditure of *Atg7*$^{\Delta/\Delta}$ mice exposed to PEG-asparaginase or PBS for 5 days. n= 12 (12 females). (C) Respiratory exchange ratio of *Atg7*$^{+/+}$ mice exposed to PEG-asparaginase or PBS for 5 days. n= 12 (12 females). (D) Respiratory exchange ratio of *Atg7*$^{\Delta/\Delta}$ mice exposed to PEG-asparaginase or PBS for 5 days. n= 12 (12 females). (E) Activity as measured by total X, Y, Z beam breaks over the course of the 5-day study. n= 12 (12 females). (F) Cumulative food intake in the CLAMS over the course of the 5-day study. n= 12 (12 females). Bar charts show mean values ± SD. Data in each graph analyzed by a two-way ANOVA (genotype x drug). Significance threshold = $p \leq 0.05$.

Supplemental Figure 5

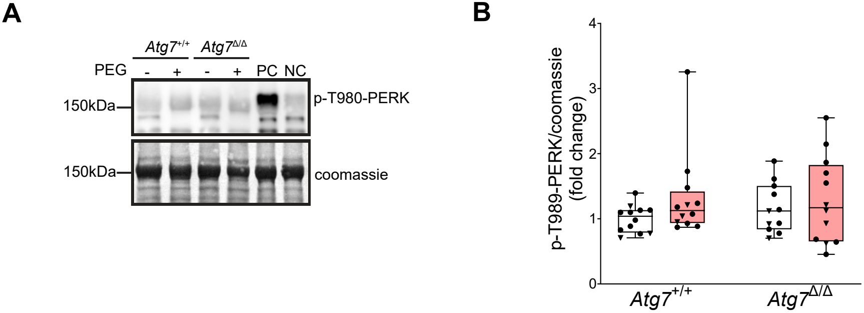

Supplemental Figure 5: Loss of *Atg7* does not activate PERK in the liver during PEG-asparaginase exposure.

(A) Representative immunoblot of hepatic p-T980-PERK and total protein as determined by coomassie staining. Liver of a wild-type mouse treated with tunicamycin used as a positive control (PC). Liver of a PERK-KO mouse treated with tunicamycin used as a negative control (NC). (B) Quantification of hepatic p-T980-PERK normalized to total protein (coomassie), expressed as fold change of *Atg7*$^{+/+}$ mice administered PBS. Box plots show median values, top and bottom hinges refer to the first and third quartiles (25th and 75th percentiles), and the ends of the whiskers mark the smallest and largest values. ●= females. ▼= males. Data in each graph analyzed by a two-way ANOVA (genotype x drug). Significance threshold = $p \leq 0.05$.

Supplemental Figure 6

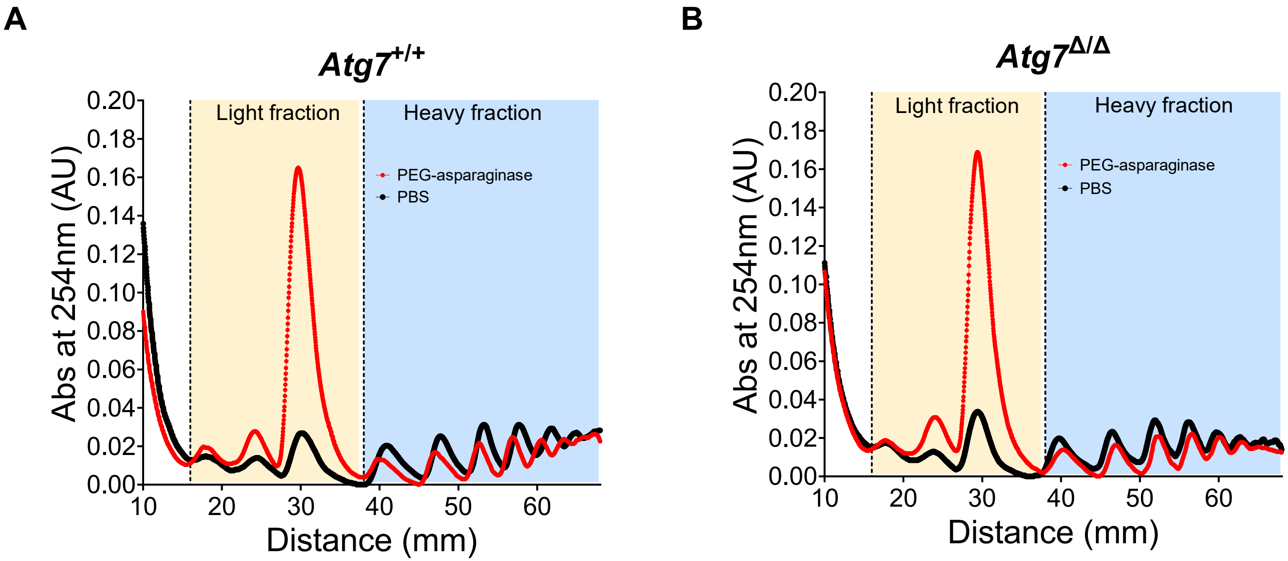

Supplemental Figure 6: Polysome profiling in the livers of *Atg7*$^{+/+}$ and *Atg7*$^{\Delta/\Delta}$ mice.

(A) Representative polysome profiles in the livers of *Atg7*$^{+/+}$ mice administered PBS or PEG-asparaginase. (B) Representative polysome profiles in the livers of *Atg7*$^{\Delta/\Delta}$ mice administered PBS or PEG-asparaginase.

Supplemental Figure 7

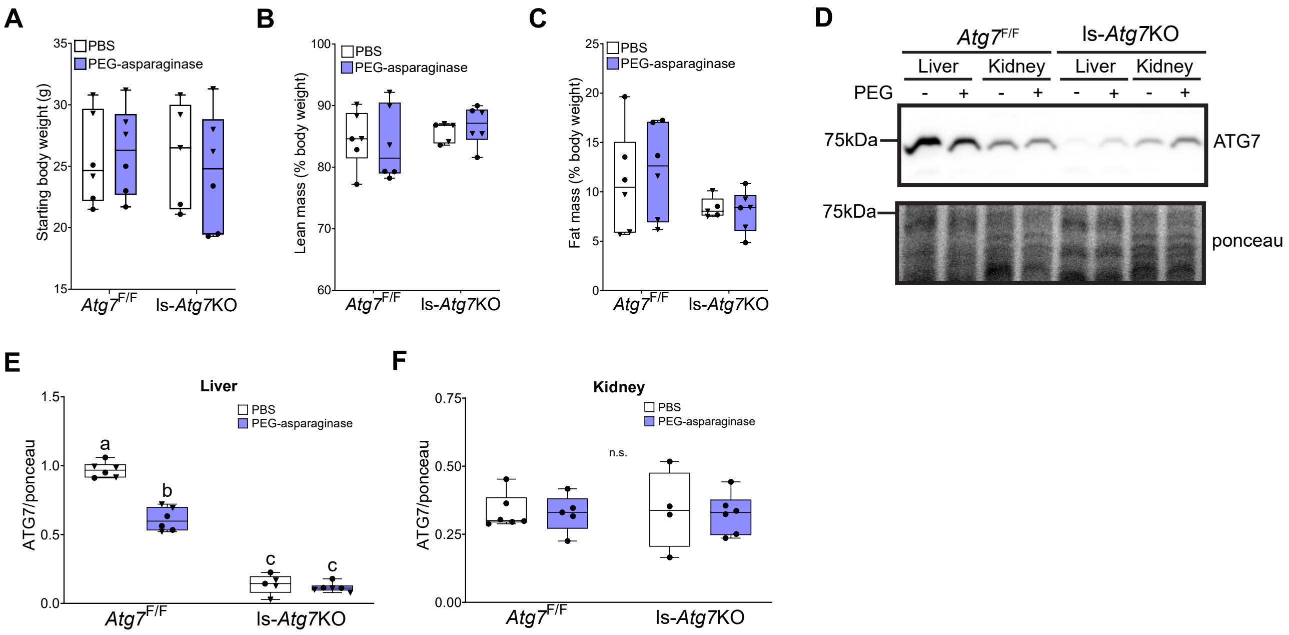

Supplemental Figure 7: Starting body composition of ls-*Atg7*KO and *Atg7*$^{F/F}$ mice and validation of liver-specific *Atg7* knockdown.

(A) Body weight at IP administration of PEG-asparaginase or PBS. n= 23 (12 males, 11 females). (B) Body lean mass, expressed as percent of initial body weight, at IP administration of PEG-asparaginase or PBS. n= 23 (12 males, 11 females). (C) Body fat mass, expressed as percent of initial body weight, at IP administration of PEG-asparaginase or PBS. n= 23 (12 males, 11 females). (D) Representative immunoblot of ATG7 protein in the liver or kidney of ls-*Atg7*KO and *Atg7*$^{F/F}$ mice. (E) Quantification of ATG7 protein in the liver normalized to total protein (ponceau). (F) Quantification of ATG7 protein in the kidney normalized to total protein (ponceau). Box plots show median values, top and bottom hinges refer to the first and third quartiles (25th and 75th percentiles), and the ends of the whiskers mark the smallest and largest values. ●= females. ▼= males. Data in each graph analyzed by a two-way ANOVA (genotype x drug). Significance threshold = $p \leq 0.05$.

Supplemental Figure 8

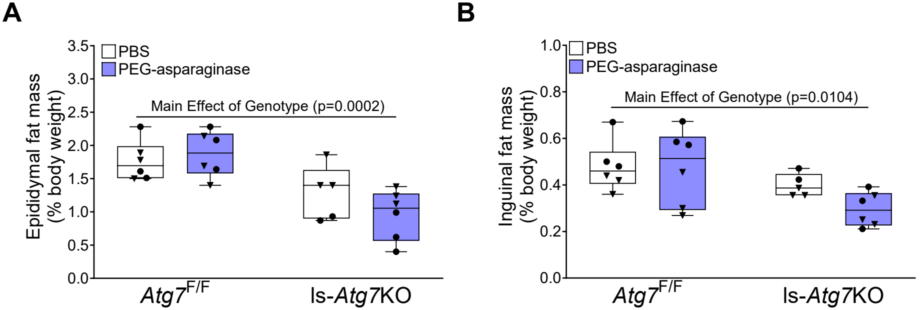

Supplemental Figure 8: Loss of hepatic *Atg7* decreases fat pad weight.

(A) Epididymal fat pad weight expressed as percent of body weight. n= 23 (12 males, 11 females). (B) Inguinal fat pad weight expressed as percent of body weight. n= 23 (12 males, 11 females). Box plots show median values, top and bottom hinges refer to the first and third quartiles (25th and 75th percentiles), and the ends of the whiskers mark the smallest and largest values. ●= females. ▼= males. Data in each graph analyzed by a two-way ANOVA (genotype x drug). Significance threshold = p≤0.05.

Supplemental Figure 9

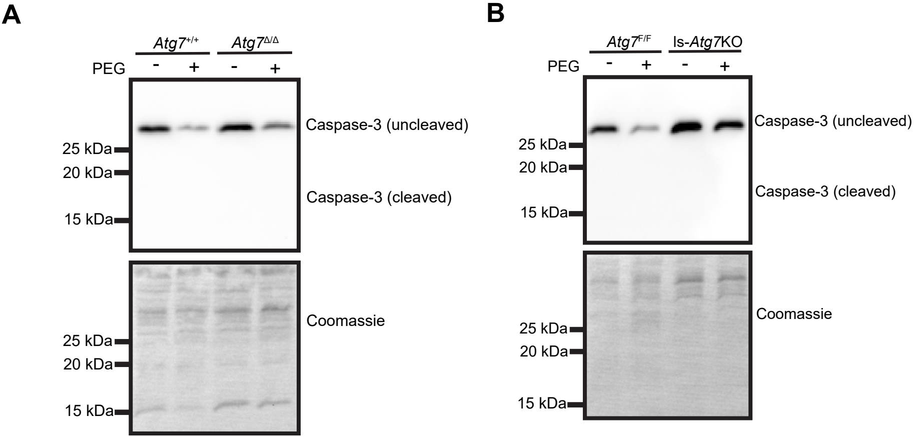

Supplemental Figure 9: Knockdown of ATG7 does not induce cleavage of caspase-3 in the liver.

(A) Immunoblot of caspase-3 in the livers of *Atg7*$^{+/+}$ and *Atg7*$^{\Delta/\Delta}$ mice exposed to PEG-asparaginase or PBS (B) Immunoblot of caspase-3 in the livers of *Atg7*$^{F/F}$ and ls-*Atg7*KO mice exposed to PEG-asparaginase or PBS control.

Supplemental Table 1. Antibodies.

Antibody	Supplier	Product number	Antibody dilution
Anti-ATG7	Sigma-Aldrich	A2856	(1:5000)
Anti-eIF2α	Santa Cruz	sc-11386	(1:2000)
Anti-p(S51)-eIF2α	Cell Signaling Technologies	3597	(1:2000)
Anti-GCN2	Cell Signaling Technologies	3302	(1:2000)
Anti-p(T899)-GCN2	Abcam	ab75836	(1:1000)
Anti-p(T980)-PERK	Cell Signaling Technologies	3179	(1:5000)
Anti-Caspase-3	Cell Signaling Technologies	9662S	(1:2000)
Peroxidase-AffiniPure Goat Anti-Rb IgG	Jackson ImmunoResearch	111-035-003	(1:20000)

Supplemental Table 2. List of primers.

	Primers 5' to 3'	
**Gene**	**Forward**	**Reverse**
Actb	GACAACTCACTCAAGATTGTCAGCAATGC	GTGGCAGTGATGGCATGGACTGTGGTC
Atf4	GAGTTTGACTTCGATGCTCTGTTTCGA	CAATTGGGTTCACTGTCTGAGGGGGCTCCTTATTAG
Atf5	TGGAGCGGGAGATCCAGTA	GACGCTGGAGACAGACGTACA
Ddit3	GAAGCCTGGTATGAGGATCTGCAGGAGGTC	CTTTGGGATGTGCGTGTGACCTCTGTTG
Asns	CTGTTACAATGGTGAAATCTACAACCACAAG	GATGAATGCAAACACCCCGTCCAGCATACAGAT
Fgf21	AGCATACCCCATCCCTGACT	AGGAGACTTTCTGGACTGCG
Gdf15	CAACCAGAGCCGAGAGGAC	TGCACGCGGTAGGCTTC
Ppp1r15a	CTTCGACTGCAGAGGCGGCTCAGATTG	GAAATGGACTGTGACTTTCTCAGCGAAGTGTAC
